# Perceptions about Healthy Eating and Emotional Factors Conditioning Eating Behaviour: A Study Involving Portugal, Brazil and Argentina

**DOI:** 10.3390/foods9091236

**Published:** 2020-09-04

**Authors:** Ana Paula Cardoso, Vanessa Ferreira, Marcela Leal, Manuela Ferreira, Sofia Campos, Raquel P. F. Guiné

**Affiliations:** 1 CI&DEI Research Centre, School of Education, Polytechnic Institute of Viseu, 3504-510 Viseu, Portugal; sofiamargaridacampos@gmail.com; 2 Department of Nutrition, School of Nursing, UFMG University, Belo Horizonte, BR 30130-100, Brazil; vanessa.nutr@gmail.com; 3 Faculty of Health Sciences, School of Nutrition, Maimonides University, Buenos Aires, AR C1405, Argentina; leal.nutricion@gmail.com; 4 UICISA:E Research Centre, School of Health, Polytechnic Institute of Viseu, 3504-510 Viseu, Portugal; mmcferreira@gmail.com; 5 CERNAS Research Centre, School of Agriculture, Polytechnic Institute of Viseu, 3504-510 Viseu, Portugal; raquelguine@esav.ipv.pt

**Keywords:** perceptions, healthy eating, emotional motivations, individual differences

## Abstract

This study analysed the perceptions about healthy eating as well as some emotional factors conditioning eating behaviour in a sample of people from Portugal, Brazil and Argentina. This is a descriptive cross-sectional study involving a non-probabilistic sample of 2501 participant. Data was collected through a questionnaire applied to adult citizens residing in their respective countries. For data analysis chi-square tests were used, and associations were evaluated by Cramer’s coefficients. Moreover, a tree classification analysis was conducted for variables related with perceptions about healthy eating and emotional conditioning of eating behaviour. The results revealed that participants’ perceptions are generally in agreement with healthy eating. However, significant differences were found between countries (*p* = 0.018) and by levels of education (*p* < 0.0005), with a more accurate perception for Portugal and at the university level. The existence of statistically significant associations between all sociodemographic variables considered and the conditioning of eating behaviour by emotional motivations should be noted. Tree classification analysis showed that the most important discriminant sociodemographic variable for perceptions about healthy eating was education, followed by professional area and country, while the most relevant discriminants for emotional conditioning of eating behaviour were country and then living environment and sex. Thus, it is important to consider these variables in initiatives that aim to promote adherence to behaviours that contribute to the health and well-being of the population.

## 1. Introduction

Human behaviour regarding food is associated with a large number of interrelated factors [1], including those of a psychological and social nature. This constitutes an inseparable act of human survival and encompasses two functions, maintain the level of nutrients necessary for the body and provide the pleasure that is derived from the act of eating through the release of serotonin and dopamine [2]. Emotions are part of the evolution of the human species and, obviously, of the development of children, adolescents and adults, constituting a fundamental part of learning. Emotions are adaptive because they prepare, predispose and guide behaviours towards positive or negative experiences, besides behaviours of survival and reproduction [3].

Human beings need a varied diet so that it is balanced and healthy [4]. This is an object of study that has been the target of an increasing number of investigations, since unhealthy eating behaviour is an important risk factor for health and mortality [5]. In the literature, there is not a consensus on organic farming being an important part of a healthful diet. Nevertheless, organic farming’s importance is increasing in the dietary patterns of some citizens in the countries studied and is considered important for the health of their population [6,7,8]. Some studies support the association between organic farming and health, like for example Costa et al. [9] report that organic farming is safer than conventional farming, because this last can cause DNA damage in people exposed to pesticides. Additionally, organic food contains higher levels of nutrients and bioactive substances that improve consumers’ health and wellness [10,11,12,13]. Organic diets have the benefit of exposing consumers to considerably lower levels of chemicals, which can cause several human diseases like cancer, autism, and infertility [14,15,16,17,18,19]. Data form pesticide residues’ monitoring clearly show that foods from organic farming have lower levels of pesticide residues when compared with foods grown in conventional farming systems [20,21].

Throughout the human developmental trajectory, all actions and thoughts are mediated by emotions. For example, eating can be motivated by positive (e.g., happiness) and negative (e.g., anger, depression) emotions, coupled with the desire to nourish [22]. Gibson [23] showed that negative emotions and depression have an influence on food. Although the typical response to stress is to eat less, some studies [24,25] have shown that eating more also appears in atypical depression.

A better understanding of the factors involved in food choices is essential to promote a healthy change in dietary behaviour [26]. Lazarevich et al. [27] conducted a study where emotional nutrition was identified as a mediating variable between depression and Body Mass Index (BMI) in young men and women. Intervention proposals in adequate nutritional education must therefore bear in mind the management of emotions and the detection of individuals vulnerable to depression and other emotional risks. While there are several papers that relate obesity and other eating disorders with emotional factors, we did not find any with the specificity of this investigation, conducted simultaneously on the different countries involved in this study.

The present study is part of the project entitled “Psychosocial motivations associated with food choices and practices” (EATMOT), which aims to analyse the different psychological and social motivations that determine people’s eating patterns, whether in relation to their eating choices or habits. It is essential to understand these factors if we intend to intervene in this area, either in terms of health promotion or in terms of disease prevention or treatment. Some of the aspects to be explored in the scope of this project include factors related to perceptions and eating habits, in areas such as emotional aspects or cultural influences.

Although eating behaviour results from a lifelong learning process, this does not invalidate that the subjects’ food preferences change over time and according to their experience and learning [4]. This reinforces the importance of understanding the influence of different types of variables, including sociodemographic ones, on eating behaviour. Hence, the main objective of this study in particular was to analyse the perceptions corresponding to healthy eating and emotional conditions linked to the eating behaviour of people from three countries (Portugal, Argentina and Brazil). It was also analysed to what extent aspects such as country of residence, age, sex, education level, living environment, marital status, or area of study or work can influence the participants’ perceptions.

## 2. Materials and Methods

### 2.1. Data Collection

The questionnaire used in this study was first validated for the Portuguese population [28,29,30] and then applied in other countries, after translation and adaptation. The questionnaire was applied in Brazil in Portuguese and translated into Spanish, following a back-translation methodology for validation. For the translation process, all the issues related to the possible cultural influences in the interpretation of the questions were verified. Moreover, in the case of Brazil, although speaking the same language, some adaptations were made in order to better suit the Brazilian way of speaking the Portuguese language.

In order to measure respondents’ perceptions about healthy eating and also about the emotional factors conditioning eating behaviour, their opinion on a set of statements was asked using a 5-point Likert scale, ranging from 1 = strongly disagree to 5 = strongly agree [31].

The questionnaire was applied, after informed consent, only to adults (18 years or older). All participants were volunteers, and their answers were collected and treated as anonymous. All ethical considerations were completely obeyed when designing the questionnaire and collecting the data, which was kept strictly confidential in such a way that none of the responses could ever be associated with the participant. The survey was approved by the Ethical Committee of Polytechnic Institute of Viseu, with reference no 04/2017.

### 2.2. Data Analysis

The data collected through the descriptive and cross-sectional study were analysed statistically, using the SPSS - Statistical Package for the Social Sciences from IBM - International Business Machines Inc. (version 24, Armonk, Nova York, EUA). Descriptive statistics was used for exploratory data analysis, and inferential statistics was also used, by means of the Chi-square test, to evaluate the association between some sociodemographic variables and the perceptions under study. Cramer’s V coefficient was used to assess the strength of the associations between the tested variables [32]. This coefficient varies from 0 to 1 and can be interpreted as V ≈ 0.1—weak association, V ≈ 0.3—moderate association, V ≈ 0.5 or over—strong association [33].

The responses obtained were transformed into new variables with two levels: value 1 (grouping the responses of agree and strongly agree) and value 0 (grouping the responses of strongly disagree and disagree). In the 1st case, that is, to measure respondents’ perceptions of healthy eating, the scale defined was as follows: “correct perceptions” (1) and “incorrect perceptions” (0); in the 2nd case, that is, to measure the emotional conditioning factors of eating behaviour, the scale was used: “conditioned” (1) and “non-conditioned” (0) eating behaviour. Responses corresponding to “no opinion” (value 3 of the scale) were not considered for this particular purpose.

The variables measuring the perceptions of healthy eating and emotional conditioning factors were analysed by tree classification analysis in order to evaluate the relative importance of the different sociodemographic variables. For this, a Classification and Regression Trees (CRT) algorithm with cross-validation was used. The classification algorithm (CRT) bases the decision process on some specified criteria, in this case defined according to the high numbers of responses. The minimum change in improvement was 0.0004, and the minimum number of cases for parent nodes was 100 and for child nodes was 50. These parameters allow the decision-making process (and this is why the classification tree can also be termed as decision tree) which happens in any of the nodes. Therefore, a parent node is a node that originates branches to other nodes, while a terminal node is that which corresponds to a stopping point. The decision to stop is derived from one of these conditions: Either the next improvement is lower that the threshold defined (0.0004), or the number of cases in the next nodes is lower than 50 (defined as minimum for child nodes).

The level of significance considered in data analysis was 5% (*p* < 0.05).

## 3. Results

### 3.1. Sample Characterization

Table 1 summarizes the sociodemographic data of the sample, that included elements from Portugal (52.2%), Argentina (20.9%) and Brazil (26.6%). There were 2501 participants in the study, of which 69.8% were female and 30.2% male, grouped by the following age groups: 43.3% of young adults (18 ≤ years ≤ 30), 39.5% of adults (31 ≤ years ≤ 50), 14.5% of senior adults (51 ≤ years ≤ 65) and 2.6% of elderly (years ≥ 66).

Most participants (59.8%) had higher education, 39.6% had completed secondary school level of education, and only 0.6% had the lowest level of education (basic school). Regarding marital status, 46.7% were single, 44.1% were married, 6.5% were divorced, and 2.8% were widowed. With regard to the environment in which they live, 84.4% lived in urban areas, 9.6% in rural areas and 6.0% in a suburban area.

The area of study or professional activity of the participants was also analysed, according to previously defined categories. It was found that 56.4% were not related to any of the listed areas and that the other respondents were distributed by the areas of health (19.8%), nutrition (10.7%), food (5.1%), sport (3.3%), psychology (2.6%) and agriculture (2.2%).

### 3.2. Perceptions About Healthy Diet

The participants revealed, in general, a perception concordant with healthy eating, as can be seen in Table 2. The high agreement of respondents in relation to some items, such as number 3 about the importance of fruits and vegetables (25.1% agree, 73.1% strongly agree), number 4 about balanced and varied diet (22.5% agree, 75.0% strongly agree), number 5 about not avoiding any foods (35.0% agree, 24.7% strongly agree) or number 9 about the value of foods from organic farming (37.4% agree, 32.6% strongly agree), is highlighted. For these items, agreement is indicative of correct perceptions about healthy diet. In the opposite way, the high disagreement with inverted items also stands out, i.e., disagreement with items that are indicative of wrong perceptions of healthy diet, such as numbers 2 (15.7% strongly disagree, 56.9% disagree) or 10 (13.3% strongly disagree, 50.7% disagree), respectively, about totally avoiding sugary or fat foods. Regarding items such as numbers 1, 6 and 8, the participants were very divided, with similar numbers for those who were for and those who were against aspects such as the diets based on counting calories, the price of healthy foods or the value of tradition to healthy dietary patterns.

Considering the influence of the seven sociodemographic variables analysed (age, sex, education, marital status, country, living environment and area of study/work) on the perceptions about healthy eating, it was observed that only in two cases there were significant differences between groups, i.e., there were statistically significant associations between perceptions and country of residence and between perceptions and education level (Table 3). The results of the Chi-square test revealed a statistically significant association between the perceptions of healthy eating and the country of residence (*p* = 0.018). For this association, the analysis of the adjusted residues indicated that the differences were in Portugal, with a more correct perception, and in Brazil, with a more incorrect perception, with values of the adjusted residues positive and greater than two. Nevertheless, despite being significant, this association is weak (V = 0.057). 

In the case of the variables, perception versus level of education (Table 3), the association was also significant (*p* < 0.0005) and weak (V = 0.092). Participants with a university degree showed a more correct perception about healthy eating than those with secondary school, according to the adjusted residues. The results further show that the perceptions about healthy eating are high for participants with the lowest level of education, basic school. This is a little surprising, and it might be due to the fact that there was not a real representative number of people with the lowest level of education, and this might be biasing the results. Hence, to confirm a possible trend of increasing education being associated with better perceptions of healthy eating, further studies should be made with more people from lower levels of education. Nevertheless, this is very difficult because the world trend, as guidance form the United Nations, is to increase the levels of education in all countries, including those under development.

**Figure 1:**
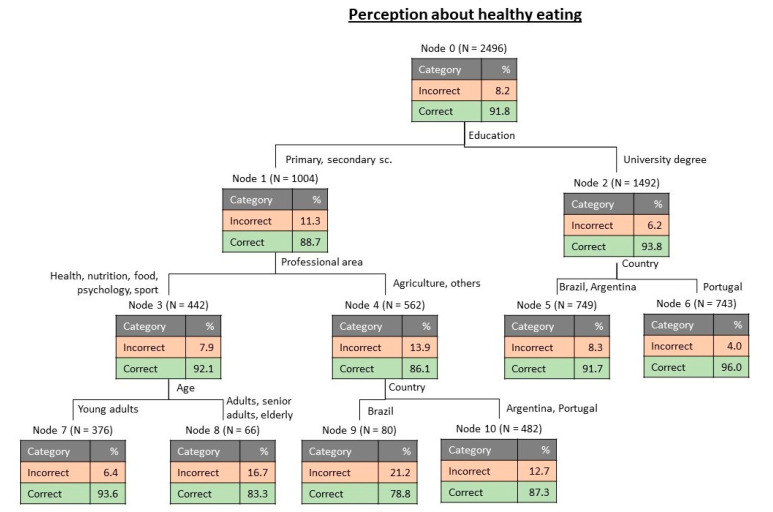
Tree classification for the perceptions about healthy eating.

The variable measuring the perceptions about healthy eating was submitted to a tree classification analysis for assessment of the relative importance of the possible influential variables. Figure 1 presents the tree obtained, and which highlights the relative importance of the sociodemographic variables to define the perceptions of the participants about a healthy diet. The estimated risk for this tree was 0.082 (with standard error 0.005) for resubstitution, and equal values were also obtained for cross-validation. The obtained tree (Figure 1) has 11 nodes, from which 6 are terminal.

Initially, i.e., for the whole number of cases, only 8.2% of the participants had an incorrect perception about healthy eating (Figure 1). The results indicated that the first discriminating variable was education, separating the participants with a university degree from the other groups, being the more educated participants those more informed about the principles of a healthy diet (only 6.2% with incorrect perception). For the participants with a university degree, the next discriminant variable was country, separating Portugal, with a lower percentage of incorrect perception, 4.0%, from the other two countries. For the participants with primary and secondary school, the next discriminant variable was professional area, separating people linked with health, nutrition, food, psychology or sport as those with better perceptions (92.1% correct). For the participants with the selected professional areas, the following discriminant was age, with young adults showing a more correct perception of healthy eating (93.6% correct). For the participants with professions linked with agriculture or other areas, country was the next discriminant, separating Brazil (78.8% correct) from the other two countries (87.3% correct).

### 3.3. Emotional Factors Conditioning the Eating Behaviour

The results in Table 4 refer to the participants’ eating behaviour according to some emotional conditioning factors. For the effect of food on coping with stress (item no 1) the participants were divided, i.e., some of them admit that food can help them deal with stress (28.8% agree, 7.1% strongly agree) while for others food does not have that outcome (35.7% disagree, 8.8% strongly disagree). However, a very expressive percentage of participants admit that food makes them feel good (item no 5: 60.7% agree, 16.7% strongly agree).

The results in Table 4 further show that most participants do not perceive food as emotional consolation (item no 8: 40.9% disagree, 22.2% strongly disagree) and regarding the role of food as a comfort for loneliness (item no 6: 45.0% disagree, 21.7% strongly disagree). For statement no 5, i.e., the act of eating as something you do when you have nothing else to occupy your time, the participants were divided, some were in favour (30.0% agree, 9.0% strongly agree), and others were against (31.7% disagree, 15.1% strongly disagree). For an expressive majority of participants, food is seen as a way to control weight (item no 2: 50.2% agree, 9.0% strongly agree), being this factor important for the emotional well-being. On the other hand, when people feel depressed, they admit having more cravings for sweets (item no 9: 36.0% agree, 20.5% strongly agree). Finally, the consumption of products (essentially beverages) with exciting properties is not so evident (item no 3: 33.4% disagree, 29.0% strongly disagree), but the ingestion of products with relaxing ability is more expressive (item no 4: 37.4% agree, 11.8% strongly agree).

The results in Table 5 demonstrate that the emotional factors influencing eating behaviour were found significantly associated with all the sociodemographic variables considered (country, age, sex, education level, living environment, marital status and area of study/work).

The results of the Chi-square (Table 5) revealed a statistically significant association between the emotional drivers of eating behaviour and country (*p* < 0.0005). The analysis of the adjusted residues indicated that the differences were for Portugal, with a lower influence when compared with Argentina and Brazil, where these factors have a little higher influence on the eating behaviour. However, this association is weak (V = 0.166). Regarding the level of education, the chi-square revealed that there were also significant differences between groups (*p* = 0.006), showing that those with secondary education were more conditioned on their eating behaviour by emotional factors, while the participants with a university degree showed the lowest impact of emotional factors. This association was, however, very weak (V = 0.064).

In relation to age (Table 5), the chi-square was equally significant (*p* < 0.0005), with young adults revealing eating behaviour a little more conditioned by emotional motivations while senior adults and the elderly were those who showed the lowest influence of emotional factors on their eating behaviours (weak association: V = 0.156). As for gender differences (*p* < 0.0005), analysis of the adjusted residues indicated that women were more susceptible to emotional conditioning of their eating behaviour as compared with men, although the association was weak (V = 0.146). Regarding living environment or marital status, the chi-square results also revealed statistically significant differences (*p* < 0.0005 in both cases). The adjusted residues confirmed that for those who lived in urban areas the emotional conditionings were considerably less expressive than for people living in suburban areas, but the association was weak (V = 0.116). In the case of widowed participants, the lowest level of influence of emotional factors on eating behaviour was observed, while the single revealed the highest level of influence. Still, the association was weak (V = 0.160). With regard to the area of studies or professional activity, there was also statistically significant differences (*p* < 0.0005), being the participants with work or studies in the field of psychology those who have eating behaviour more conditioned by emotional aspects (weak association: V = 0.154).

**Figure 2:**
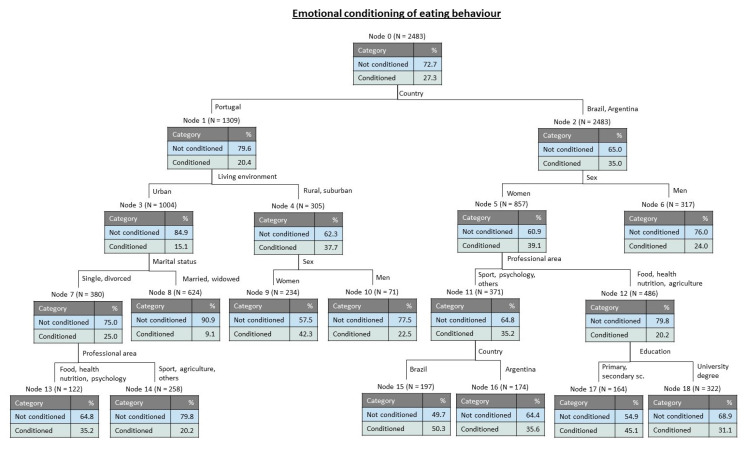
Tree classification for the emotional conditionings of eating behaviour.

Moreover, the variable measuring the emotional conditioning of eating behaviour was submitted to a tree classification analysis and the obtained results are shown in Figure 2. The estimated risk for this tree was 0.273 (with standard error 0.009) for resubstitution and 0.281 (with standard error 0.009) for cross-validation. The obtained tree has 18 nodes, being 9 of them terminal. The results showed that for the whole number of cases (node 0) 72.7% of participants have eating behaviours not conditioned by emotional factors. For this variable, the first discriminant was country, separating Portugal (79.6% not conditioned) from Brazil and Argentina. While for Portugal the next discriminant was living environment (Urban: 15.1% conditioned), for the other countries the next discriminant was sex (women more conditioned by emotional factors than men: 39.1% and 24.0%, respectively). For the women in Brazil and Argentina with professions or studies related to food, nutrition or agriculture, education was the following discriminant, separating those with a university degree as being less susceptible to condition their eating behaviours in function of emotional aspects (31.1% conditioned against 45.1% for those with less education). For participants from Portugal living in urban areas, the next discriminant was marital status, separating the single and divorced as being more conditioned (25.0) than the married or widowed (9.1%). For these groups of single and divorced people, the next discriminant was professional area, with participants linked with food, health, nutrition and psychology showing more conditioned eating habits (35.2%).

## 4. Discussion

The results revealed that the participants’ perceptions about healthy eating were, in general, correct. Significant differences were found by country and level of education, with adult citizens residing in Portugal and those with higher education showing a more correct perception of what healthy eating should be. These results are in line with those of the study by Ferrão et al. [30]. They are also consistent with the results of Lê et al. [34] who showed an association between a high educational level and adherence to a healthy diet. High agreement regarding the importance of fruits and vegetables in the diet as well as of a varied and balanced diet or disagreement as to the avoidance of sugary or fatty foods in the three countries was observed. This generalized opinion can be explained by the European influence shared by these countries, despite the cultural specificities of each one, and the well spread information about the health benefits of fruits and vegetables [35,36] while fats, particularly saturated fat, and sugars may contribute for important health morbidities like diabetes, heart diseases and obesity [37,38,39]. In relation to the differences between countries in particular, the more correct perception of what is the healthy diet in Portugal may be associated with the Mediterranean diet [40] that characterizes the gastronomic tradition of Portugal, in contrast to Brazil and Argentina. There is not one global definition of healthy diet; however, as discussed earlier, there are worldwide recommendations and recognition of some unhealthy foods, which leaves some space for country differences. The correct perception of healthy eating, associated with a greater nutritional knowledge, is recognized as an important factor in the promotion of adequate eating behaviours, as some scientific evidence suggests [41,42]. This emphasizes the relevance of training aimed at increasing food literacy, with a view to better stimulate suitable food choices in the general population. For this purpose, additional studies must be carried out in order to better understand how information should be provided in each of the participating countries. It is suggested that the results of this work be discussed among health professionals (including those linked with psychology) to analyse the best way of action according to the specificities of each country regarding health policies, health care systems, educational systems and their coordination towards a common objective. This must be done separately in each of the countries, given their social, cultural and political differences. These differences should be communicated to the proper organisms to include them in education campaigns, designed in the different countries according to their specificity. Regarding the emotional factors conditioning eating behaviour, the need for emotional management is transversal to the different cultures. Therefore, the organizers of health-promoting initiatives in each of these countries should organize dynamic and interactive sessions, in which participants have the opportunity to exchange their own experiences and recognize the association between emotions and eating behaviours, as well as the importance of an adequate emotional eating management, in order to develop emotional skills to achieve a healthier diet. More specifically, some authors [25,27,43] highlight the application of 3rd Generation Therapies to regulate emotions as a means of balancing dietary changes. The most referred techniques are cognitive-behavioural psychotherapies, mindfulness and acceptance and commitment, as they are those that have the greatest impact and that present the best results.

In this study, statistically significant associations were found between the emotional motivations for eating behaviour and all sociodemographic variables (country, living environment, age, sex, educational level, marital status and area of study/work). In the investigation by Chambers et al. [44], the results revealed that different emotional motivations are related to food, namely, eating to relieve stress, weight control, emotional comfort and eating sweets to relieve depressive states. The same authors state that food choices and their quantity and quality vary according to the individual’s particular characteristics and according to a specific emotional status. However, Ashurst et al. [45] mention that, despite the importance of affective processes in eating behaviour, it is still difficult to predict how emotions affect the act of eating. They highlight the importance of individual differences and emphasize that previous research did not focus much on the overlapped variability of changes in eating behaviour induced by emotions, justified, on one hand, by the differences between the individuals and, on the other hand, by their emotions according to a particular moment or experience.

Our results showed that food can serve as emotional consolation, help deal with stress and fight loneliness or boredom. In an investigation carried out with university students, Bennett et al. [46] found that food is often used as a way to distract attention from negative emotions. According to Boggiano [47], eating behaviour that deals with negative emotions is called “emotional eating” and individuals with emotional eating behaviour eat for reasons other than physiological needs. These individuals generally continue to eat to achieve the balance of the homeostatic shift of energy in a positive direction, especially in case of emotional situations.

Bennett et al. [46] found that when participants reported negative emotions, they were more likely to consume meat/proteins and sweets. However, our results are not so conclusive on this, because only a small percentage of participants assumed that when they felt depressed their cravings for sweets increased.

Young adults, women, residents in Brazil or Argentina, those who live in rural areas, those who are single or divorced, those who have secondary education, or whose area of study or work is related with nutrition, psychology, food or health are the ones that evidence a higher degree of conditioning in their eating behaviour according to emotional aspects. These results are, to some extent, in line with those of the research by Bartkiene et al. [48], which indicated that age, education level and sex are associated with emotional motivations for eating. The works by Bennett et al. [46] and Guiné et al. [49] also reported gender differences in the emotional factors driving eating behaviour.

Significant differences were found in the perceptions about healthy eating for countries and education levels, with more correct perceptions for people from Portugal and with a university degree. A plausible explanation for these differences has to do with the gastronomic tradition of each country, with emphasis on the prevalence of the Mediterranean diet in Portugal, as discussed earlier, considered one of the healthiest [50]. Regarding the educational level, attending higher education drives the students to a constant search for new information and development of the critical thinking, and this allows greater awareness also about the benefits of healthy eating and the constant need to search for more correct information about food and its effects [51,52].

Other variables, like area of study/work, living environment or marital status, showed also differences in the way emotional aspects influence eating behaviour. It has been referred that different social statuses are linked to different behaviours on several aspects and also linked with food and eating habits. The cultural context proved to be a determining factor in the selection of the type of food consumed and quantities ingested, which is in line with the investigation by Castro et al. [53] who compared the eating behaviours of French, American and German university students and found that there were marked differences between these cultures in terms of quantity, composition, diurnal rhythm and pattern of food intake between the different cultures.

According to literature, a more personalized analysis is required in the design of proposals that aim to promote adherence to healthy eating behaviours [48]. Decision-makers and professionals responsible for organizing health-promoting initiatives should therefore seek to increase citizens’ food literacy levels and address the diversity of individual variables, such as sex, age, education level, living environment and culture of origin, in the design of training proposals, in order to undertake more effective actions in adhering to behaviours that contribute to the health and well-being of the population.

## 5. Conclusions and Limitations

The results of this study indicated that, in general, the participants’ perceptions about healthy eating are in line with the preconized principles of a healthy diet, particularly regarding the importance of consuming fruits and vegetables, of practicing a balanced, varied and complete diet, and of consuming foods from organic farming. Significant differences were found in the perceptions about healthy eating for countries and education levels, with more correct perceptions for people from Portugal and with a university degree.

Concerning the emotional factors and the way they influence eating patterns, a low level of influence was found, particularly in aspects related with the role of food as consolation or as a way to deal with stress. The emotional conditionings of eating behaviour were found to significantly vary according to all sociodemographic variables tested.

Tree classification analysis revealed that the most important discriminant for perceptions about healthy eating was education, followed by professional area and country, while the most relevant discriminants for emotional conditioning of eating behaviour were country followed by living environment and sex.

One of the limitations of this study derives from the fact that it covers a sample that, although vast, is not probabilistic, therefore does not give sufficient guarantees to be representative of the entire population. Additionally, derived from being a convenience sample, the representativeness of all groups was not even, as for example for sex or education levels. Therefore, these results must be understood as exploratory and further studies should be undertaken for proper generalization of the results. Another limitation has to do with the fact that the questionnaire was only validated for Portugal, despite having the collaboration of researchers from the other countries involved in the work of translation and adaptation to the cultural context.

## Figures and Tables

**Table 1:** Sociodemographic characterization of the sample (N = 2501).

Variable	Groups	%	Variable	Groups	%
Age ^1^	Young adults	43.4	Country	Argentina	20.9
	Adults	39.5		Brazil	26.6
	Senior adults	14.5		Portugal	52.5
	Elderly	2.6	Living environment	Rural	9.6
Sex	Women	69.8		Urban	84.4
	Men	30.2		Suburban	6.0
Education	Basic school	0.6	Area of study/work	Nutrition	10.7
	Secondary school	39.6		Food	5.1
	University degree	59.8		Agriculture	2.2
Marital status	Single	46.7		Sport	3.3
	Married	44.1		Psychology	2.6
	Divorced	6.5		Health	19.8
	Widowed	2.8		Others	56.4

^1^ Young adults: 18 ≤ years ≤ 30, Adults: 31 ≤ years ≤ 50, Senior adults: 51 ≤ years ≤ 65, Elderly: years ≥ 66.

**Table 2:** Perceptions about healthy eating (Scale from 1 = strongly disagree to 5 = strongly agree).

Perceptions About Healthy Eating	Percentage of Answers According to Scale Points				
	1 (%)	2 (%)	3 (%)	4 (%)	5 (%)
1. Healthy eating is based on counting calories	17.2	27.9	18.2	25.6	11.1
2. We should never consume sugary products	15.7	56.9	14.2	10.6	2.6
3. Fruits and vegetables are important for a healthy diet	0.6	0.2	1.1	25.1	73.1
4. A healthy diet must be balanced, varied and complete	0.6	0.2	1.8	22.5	75.0
5. We can eat everything as long as it is in small quantities	4.4	24.4	11.5	35.0	24.7
6. I believe that a healthy diet is expensive	16.8	28.4	21.9	24.1	8.8
7. In my opinion it is strange that some people have cravings for sweets	31.7	50.7	12.4	4.0	1.2
8. I believe that tradition is very important for healthy eating	10.8	25.0	25.1	30.3	8.9
9. I believe that food coming from organic farming is healthier	1.5	5.7	22.8	37.4	32.6
10. We should never consume high fat foods	13.3	50.7	17.9	13.5	4.6

**Table 3:** Perceptions about healthy eating according to country and level of education.

Variable	Perception		Chi-Square Test	Cramer’s Coefficient
	Correct	Incorrect		
	(%)	(%)	*p*	V
*Country*			0.018	0.057
Argentina	90.8	9.2		
Brazil	89.7	10.3		
Portugal	93.2	6.8		
*Education*			<0.0005	0.092
Basic school	93.3	6.7		
Secondary school	88.7	11.3		
University degree	93.8	6.2		

**Table 4:** Emotional factors conditioning the eating behaviour (Scale from 1 = strongly disagree to 5 = strongly agree).

Emotional Factors Conditioning the Eating Behaviour	Percentage of Answers According to Scale Points				
	1 (%)	2 (%)	3 (%)	4 (%)	5 (%)
1. Food helps me deal with stress	8.8	35.7	19.7	28.8	7.1
2. I usually eat food that helps me control my weight	3.6	16.2	21.0	50.2	9.0
3. I often consume products that keep me awake and alert (such as coffee, cola, energy drinks)	29.0	33.4	11.1	19.8	6.8
4. I often consume products that help me relax (such as, for example, infusions of herbs with calming properties)	10.4	23.2	17.2	37.4	11.8
5. Food makes me feel good	1.4	4.3	17.0	60.7	16.7
6. When I feel alone, I comfort myself/take refuge in food	21.7	45.0	15.5	12.9	4.9
7. I eat more when I have nothing to do	15.1	31.7	14.2	30.0	9.0
8. For me, food serves as an emotional consolation	22.2	40.9	16.6	15.2	5.2
9. When I’m depressed, I have more cravings for sweets	20.5	36.0	13.7	20.4	9.4

**Table 5:** Emotional conditionings of eating behaviour according to sociodemographic variables.

Variable	Eating Habits		Chi-Square Test	Cramer’s Coefficient
	Conditioned	Not Conditioned		
	(%)	(%)	*p*	V
Country			<0.0005	0.166
Argentina	33.2	66.8		
Brazil	36.4	63.6		
Portugal	20.4	79.6		
Education			0.006	0.064
Basic school	26.7	73.3		
Secondary school	30.8	69.2		
University degree	25.0	75.0		
Age ^1^			<0.0005	0.156
Young adults	34.0	66.0		
Adults	25.3	74.7		
Senior adults	15.2	84.8		
Elderly	12.5	87.5		
Sex			<0.0005	0.146
Women	31.6	68.4		
Men	17.4	82.6		
Marital status			<0.0005	0.160
Single	34.1	65.9		
Married	21.0	79.0		
Divorced	30.4	69.6		
Widowed	7.2	92.8		
Living environment			<0.0005	0.116
Rural	37.7	62.3		
Urban	25.1	74.9		
Suburban	41.6	58.4		
Area of study/work			<0.0005	0.154
Nutrition	37.6	62.4		
Food	37.8	62.2		
Agriculture	20.4	79.6		
Sport	26.8	73.2		
Psychology	46.2	53.8		
Health	32.2	67.8		
Others	22.1	77.9		

^1^ Young adults: 18 ≤ years ≤ 30, Adults: 31 ≤ years ≤ 50, Senior adults: 51 ≤ years ≤ 65, Elderly: years ≥ 66.

## References

[1] Köster E.P. (2009). Diversity in the determinants of food choice: A psychological perspective. Food Qual. Prefer..

[2] Lent R. (2004). Cem Bilhões de Neurónios: Conceitos Fundamentais de Neurociencia.

[3] Fonseca V. (2016). Importância das emoções na aprendizagem: Uma abordagem neuropsicopedagógica. Rev. Psicopedag..

[4] Ogden J. (2002). The Psychology of Eating: From Healthy to Disordered Behavior.

[5] GHDX (2017). Global Burden of Disease Study 2017.

[6] Annunziata A., Vecchio R. (2016). Organic farming and sustainability in food choices: An analysis of consumer preference in southern Italy. Agric. Sci. Procedia.

[7] Danner H., Menapace L. (2020). Using online comments to explore consumer beliefs regarding organic food in German-speaking countries and the United States. Food Qual. Prefer..

[8] Rana J., Paul J. (2017). Consumer behavior and purchase intention for organic food: A review and research agenda. J. Retail. Consum. Serv..

[9] Costa C., García-Lestón J., Costa S., Coelho P., Silva S., Pingarilho M., Valdiglesias V., Mattei F., Dall’Armi V., Bonassi S. (2014). Is organic farming safer to farmers’ health? A comparison between organic and traditional farming. Toxicol. Lett..

[10] Kamp M.E., Saridakis I., Verkaik-Kloosterman J. (2019). Iodine content of semi-skimmed milk available in the Netherlands depending on farming (organic versus conventional) and heat treatment (pasteurized versus UHT) and implications for the consumer. J. Trace Elem. Med. Biol..

[11] Armesto J., Rocchetti G., Senizza B., Pateiro M., Barba F.J., Domínguez R., Lucini L., Lorenzo J.M. (2020). Nutritional characterization of *Butternut squash* (*Cucurbita moschata* D.): Effect of variety (Ariel vs. Pluto) and farming type (conventional vs. organic). Food Res. Int..

[12] Martí R., Leiva-Brondo M., Lahoz I., Campillo C., Cebolla-Cornejo J., Roselló S. (2018). Polyphenol and l-ascorbic acid content in tomato as influenced by high lycopene genotypes and organic farming at different environments. Food Chem..

[13] Reeve J.R., Hoagland L.A., Villalba J.J., Carr P.M., Atucha A., Cambardella C., Davis D.R., Delate K., Sparks D.L. (2016). Chapter six—Organic farming, soil health, and food quality: Considering possible links. Advances in Agronomy.

[14] Hyland C., Bradman A., Gerona R., Patton S., Zakharevich I., Gunier R.B., Klein K. (2019). Organic diet intervention significantly reduces urinary pesticide levels in U.S. children and adults. Environ. Res..

[15] Melgarejo M., Mendiola J., Koch H.M., Moñino-García M., Noguera-Velasco J.A., Torres-Cantero A.M. (2015). Associations between urinary organophosphate pesticide metabolite levels and reproductive parameters in men from an infertility clinic. Environ. Res..

[16] Kuang L., Hou Y., Huang F., Hong H., Sun H., Deng W., Lin H. (2020). Pesticide residues in breast milk and the associated risk assessment: A review focused on China. Sci. Total Environ..

[17] Sabarwal A., Kumar K., Singh R.P. (2018). Hazardous effects of chemical pesticides on human health–Cancer and other associated disorders. Environ. Toxicol. Pharmacol..

[18] Martin F.L., Martinez E.Z., Stopper H., Garcia S.B., Uyemura S.A., Kannen V. (2018). Increased exposure to pesticides and colon cancer: Early evidence in Brazil. Chemosphere.

[19] Philippat C., Barkoski J., Tancredi D.J., Elms B., Barr D.B., Ozonoff S., Bennett D.H., Hertz-Picciotto I. (2018). Prenatal exposure to organophosphate pesticides and risk of autism spectrum disorders and other non-typical development at 3 years in a high-risk cohort. Int. J. Hyg. Environ. Health.

[20] Forman J., Silverstein J. (2012). Organic foods: Health and environmental advantages and disadvantages. Pediatrics.

[21] USDA (2016). Pesticide Data Program.

[22] Jackson B., Cooper M.L., Mintz L., Albino A. (2003). Motivations to eat: Scale development and validation. J. Res. Personal..

[23] Gibson E.L. (2006). Emotional influences on food choice: Sensory, physiological and psychological pathways. Physiol. Behav..

[24] Gold P.W., Chrousos G.P. (2002). Organization of the stress system and its dysregulation in melancholic and atypical depression: High vs low CRH/NE states. Mol. Psychiatry.

[25] Paans N.P.G., Gibson-Smith D., Bot M., van Strien T., Brouwer I.A., Visser M., Penninx B.W.J.H. (2019). Depression and eating styles are independently associated with dietary intake. Appetite.

[26] Love H., Bhullar N., Schutte N.S. (2019). Psychological aspects of diet: Development and validation of three measures assessing dietary goal-desire incongruence, motivation, and satisfaction with dietary behavior. Appetite.

[27] Lazarevich I., Camacho M.E.I., Velázquez-Alva M.d.C., Zepeda M. (2016). Relationship among obesity, depression, and emotional eating in young adults. Appetite.

[28] Ferrão A.C., Guine R.P.F., Correia P.M.R., Ferreira M., Lima J.D. (2019). Development of a questionnaire to assess people’s food choices determinants. Curr. Nutr. Food Sci..

[29] Ferrão A.C., Correia P., Ferreira M., Guiné R.P.F. (2019). Perceptions towards healthy diet of the portuguese according to area of work or studies. Zdr Varst.

[30] Ferrão A.C., Guiné R.P.F., Correia P., Ferreira M., Cardoso A.P., Duarte J., Lima J. (2018). Perceptions towards a healthy diet among a sample of university people in Portugal. Nutr. Food Sci..

[31] Likert R. (1932). A technique for the measurement of attitudes. Arch. Psychol..

[32] Pestana M.H., Gageiro J.N. (2014). Análise de Dados para Ciências Sociais—A complementaridade do SPSS.

[33] Witten R., Witte J. (2009). Statistics.

[34] Lê J., Dallongeville J., Wagner A., Arveiler D., Haas B., Cottel D., Simon C., Dauchet L. (2013). Attitudes toward healthy eating: A mediator of the educational level-diet relationship. Eur. J. Clin. Nutr..

[35] Thow A.M., Verma G., Soni D., Soni D., Beri D.K., Kumar P., Siegel K.R., Shaikh N., Khandelwal S. (2018). How can health, agriculture and economic policy actors work together to enhance the external food environment for fruit and vegetables? A qualitative policy analysis in India. Food Policy.

[36] Sharps M., Robinson E. (2016). Encouraging children to eat more fruit and vegetables: Health vs. descriptive social norm-based messages. Appetite.

[37] Park H., Yu S. (2019). Policy review: Implication of tax on sugar-sweetened beverages for reducing obesity and improving heart health. Health Policy Technol..

[38] Carbone S., Lavie C.J., Elagizi A., Arena R., Ventura H.O. (2020). The impact of obesity in heart failure. Heart Fail. Clin..

[39] DiNicolantonio J.J., Lucan S.C., O’Keefe J.H. (2016). The evidence for saturated fat and for sugar related to coronary heart disease. Prog. Cardiovasc. Dis..

[40] Teixeira B., Afonso C., Sousa A.S., Guerra R.S., Santos A., Borges N., Moreira P., Padrão P., Amaral T.F. (2019). Adherence to a mediterranean dietary pattern status and associated factors among Portuguese older adults: Results from the nutrition up 65 cross-sectional study. Nutrition.

[41] Dammann K.W., Smith C. (2011). Food-related environmental, behavioral, and personal factors associated with body mass index among urban, low-income African-American, American Indian, and Caucasian women. Am. J. Health Promot..

[42] Rustad C., Smith C. (2013). Nutrition knowledge and associated behavior changes in a holistic, short-term nutrition education intervention with low-income women. J. Nutr. Educ. Behav..

[43] Strien T. (2018). Causes of emotional eating and matched treatment of obesity. Curr. Diab. Rep..

[44] Chambers D., Phan U.T.X., Chanadang S., Maughan C., Sanchez K., Di Donfrancesco B., Gomez D., Higa F., Li H., Chambers E. (2016). Motivations for Food Consumption during specific eating occasions in Turkey. Foods.

[45] Ashurst J., van Woerden I., Dunton G., Todd M., Ohri-Vachaspati P., Swan P., Bruening M. (2018). The association among emotions and food choices in first-year college students using mobile-ecological momentary assessments. BMC Public Health.

[46] Bennett J., Greene G., Schwartz-Barcott D. (2013). Perceptions of emotional eating behavior. A qualitative study of college students. Appetite.

[47] Boggiano M.M. (2016). Palatable eating motives scale in a college population: Distribution of scores and scores associated with greater BMI and binge-eating. Eat. Behav..

[48] Bartkiene E., Steibliene V., Adomaitiene V., Juodeikiene G., Cernauskas D., Lele V., Klupsaite D., Zadeike D., Jarutiene L., Guiné R.P.F. (2019). Factors affecting consumer food preferences: Food taste and depression-based evoked emotional expressions with the use of face reading technology. Bio. Med. Res. Int..

[49] Guiné R., Ferrão A.C., Ferreira M., Correia P., Cardoso A.P., Duarte J., Rumbak I., Shehata A.-M., Vittadini E., Papageorgiou M. (2019). The motivations that define eating patterns in some Mediterranean countries. Nutr. Food Sci..

[50] Hidalgo-Mora J.J., García-Vigara A., Sánchez-Sánchez M.L., García-Pérez M.-Á., Tarín J., Cano A. (2020). The Mediterranean diet: A historical perspective on food for health. Maturitas.

[51] Latha S. (2020). Vuca in engineering education: Enhancement of faculty competency for capacity building. Procedia Comput. Sci..

[52] Bezanilla M.J., Fernández-Nogueira D., Poblete M., Galindo-Domínguez H. (2019). Methodologies for teaching-learning critical thinking in higher education: The teacher’s view. Think. Ski. Creat..

[53] Castro J.M., Bellisle F., Feunekes G.I.J., Dalix A.-M., De Graaf C. (1997). Culture and meal patterns: A comparison of the food intake of free-living American, Dutch, and French students. Nutr. Res..

